# The autophagy gene product BEC-1 supports normal aging and neurodevelopment in *Caenorhabditis elegans*: Integration

**DOI:** 10.17912/micropub.biology.000102

**Published:** 2019-06-15

**Authors:** Nicholas Ashley, Andrea M Holgado

**Affiliations:** 1 St. Edward's University, Department of Biological Sciences, Austin, TX 78704

**Figure 1.**
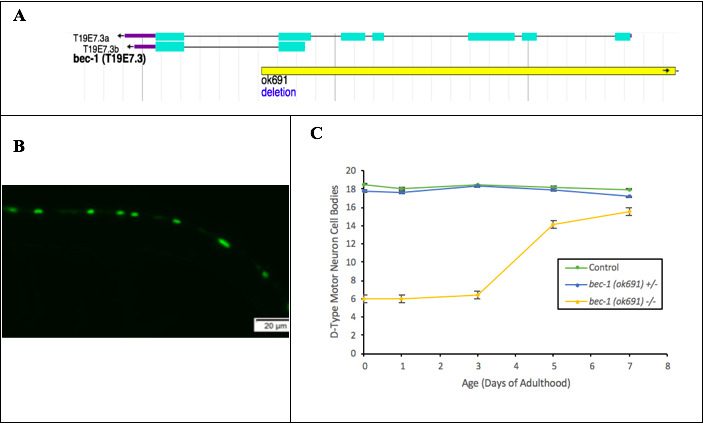
**Homozygous *bec-1 (ok691)* mutants show a delay in the development of VD motor neurons.** (A) Schematic of *bec-1* gene shown in light blue and *bec-1* (*ok691)* deletion mutation in yellow (wormbase.org). (B) D-type motor neurons marked by GFP in homozygous *bec-1* *(ok691)* mutant nematodes on day 5 of adulthood. (C) Quantification of D-type motor neuron cell bodies on days 0,1,3,5,and 7 of adulthood was recorded. Data plotted are mean ± 1 SEM, n=60. Statistical analysis was performed using a non-parametric Kruskal-Wallis (p< .001).

## Description

Aging can be defined by many different physiological pathways that emphasize changes in a variety of cells and tissues. Disease and death are often results of aging (Viña et al., 2013). However, specific mechanisms in different cell types offer multiple hypotheses regarding the definition of aging (Viña et al., 2007). Although some researchers target specific aspects of physiological processes as major contributors to aging, such as free radical theory (Harman, 2003), inflammation theory (Chung et al., 2001), mitochondrial theory (Cadenas and Davies, 2000; Sastre et al., 2000), immunologic theory (Franceschi et al., 2000), current hypotheses suggest that aging results from alterations of multiple systems working at the same time (Weinert and Timiras, 2003). Central Nervous System neural networks have been identified as particularly important for understanding cellular processes that cause aging, which is partly characterized by cell death (Yonekawa and Thorburn, 2013).

Macroautophagy (hereon referred as Autophagy) is a cellular housekeeping mechanism that uses a double membrane to target and engulf cell products forthe formation of autophagosomes. These double membrane organelles then fuse to lysosomes where cell products aredegraded and recycled (Nakamura and Yoshimori, 2018). Reports show that autophagy plays an important role in pathogen defense, development, starvation adaptations, and aging (Mizushima et al., 2008).Analysis of autophagy mutants in *C. elegans* revealed that *bec-1/Atg6/Beclin 1* is essential for dauer development, a quiescent state that survives harsh conditions such as lack of nutrients, high nematode density, and high temperatures by inducing autophagy (Meléndez et al., 2003; Meléndez and Levine 2009).

To further investigate the phenotypes associated with the *bec-1(ok691)* mutation, we studied nematodes possessing a 3000 base pair deletion mutation (allele *ok691*) in the *bec-1* locus (Figure 1A). Previous reports show that the *bec-1(ok691)* mutation is lethal, however a small proportion of homozygous *bec-1(ok691)* animals reach adulthood due to maternal effect, but do not reproduce due to sterility (Melendez and Levine 2009). Our analysis of survival throughout adulthood shows thatlifespan is significantly reduced in homozygous *bec-1(ok691)* mutants (Ashley and Holgado, 2019a). In contrast, heterozygous *bec-1(ok691)* mutant lifespan is not significantly different from control animals, ruling out previously discovered haploid insufficiency effects of Beclin 1 on autophagy in *C. elegans* (Sinha and Levine, 2008).

Previous research has found increased lifespans of *daf-2* mutant *C. elegans*, a genetic lesion that affects the insulin-like signaling pathway and promotes constitutive dauer formation (Kimura et al. 1997). Furthermore, this *daf-2*-dependent extension of lifespan was eliminated in *bec-1(ok691)*; *daf-2(e1370)* double mutants. Together, these findings support the notion that the autophagy gene product BEC-1 is needed for the *daf-2*-dependent lifespan extension of *C. elegans*. Additionally, double mutants *Atg-7* and *bec-1(ok691)* also show a decrease in lifespan in nematodes with inherent dietary restriction (Tóth et al., 2008).

The loss of autophagy function in the motor cortex has been associated with progression of neurodegenerative symptoms in Parkinson’s disease (Kaila and Lang 2015; Fahn et al. 2004). We showed locomotion in *C. elegans* mutants for *bec-1* had significant defects (Ashley and Holgado, 2019b). These defects in locomotion suggest a role of autophagy controlling behaviors that are neuronal dependent.

Considering our findings of defective locomotion and lifespan in a*bec-1(ok691)* mutant background, we proceeded our research by assessing density of motor neurons in the ventral nerve cord of *C. elegans*. After analyzing possible effects of the *bec-1(ok691)* mutation on neuronal density, we followed the transgene *juIs76* as it produced GFP marked D-type motor neurons (Figure 1 B). These studies showed a delay in development of ventral D-type motor neurons in *bec-1(ok691)* homozygous mutants (Figure 1 C). Maturation and development of VD motor neurons to the levels of controls were seen on day 5 of adulthood in *bec-1(ok691)* homozygous mutants (Ashley and Holgado, 2019c).

Previous research has found lineage timing of GABAergic (VD) motor neuron differentiation in *C. elegans* to occur before animals reach adulthood (Jin et al., 1994). However, our discovery in delayed lineage timing of VD motor neurons suggests a potential role of BEC-1 in neurodevelopment. This is consistent with findings in mouse models, where ortholog *Beclin 1* plays an essential role in cell differentiation during development (Cecconi and Levine, 2008). Instead of observing rapid neurodegeneration of VD motor neurons, resulting from the *bec-1(ok691)* mutation, we observed a rapid decrease in lifespan as VD motor neurons were differentiated. There is additional evidence that suggests autophagy’s role in mechanisms of cell editing in early developmental stages of *C. elegans* (Di Bartolomeo et al., 2010). These conclusions should be considered as preliminary as we havenot verified by an alternative line of investigation (e.g., a secondallele or transgene rescue) that the observed phenotypes are specific to *bec-1(ok691).* Specific mechanisms in which neurodegeneration act are still unknown. Future investigations observing the status of autophagy in *bec-1(ok691)* mutants may contribute novel knowledge into suggesting how autophagy may influence a delay in development of VD motor neurons or uncover pieces of the mechanism causing neurodegeneration in similar autophagy mutants.
